# Fish-Free Diet in Patients with Phenylketonuria Is Not Associated with Early Atherosclerotic Changes and Enhanced Platelet Activation

**DOI:** 10.1371/journal.pone.0135930

**Published:** 2015-08-20

**Authors:** Patrik Htun, Jens Nee, Ursula Ploeckinger, Klaus Eder, Tobias Geisler, Meinrad Gawaz, Wolfgang Bocksch, Suzanne Fateh-Moghadam

**Affiliations:** 1 Zentrale Notaufnahme und Medizinische Klinik IV, Zentralklinikum Augsburg, Germany; 2 Medizinische Klinik mit Schwerpunkt Nephrologie und Intensivmedizin, Charite-Universitaetsmedizin Campus Virchow-Klinikum, Humboldt-Universitaet zu Berlin, Germany; 3 Interdisziplinaeres Stoffwechsel-Centrum, Charite-Universitaetsmedizin Campus Virchow-Klinikum, Humboldt-Universitaet zu Berlin, Germany; 4 Institut für Tierernährung und Ernährungsphysiologie, Justus-Liebig-Universität Giessen, Germany; 5 Medizinische Klinik III, Abteilung für Kardiologie und Kreislauferkrankungen, Eberhard-Karls Universitaet Tuebingen, Germany; University Hospital Medical Centre, GERMANY

## Abstract

**Background and Purpose:**

Since patients with phenylketonuria (PKU) have to follow a lifelong restriction of natural protein to lower phenylalanine-intake, they never eat fish. This diet may lead to a chronic deficit of omega-3 and omega-6 fatty acids with the risk of early atherosclerotic changes. The aim of the study was to analyse the fatty acid profile of PKU patients and to correlate the results with surrogate markers of early atherosclerotic changes [enhanced carotid intima media thickness (CIMT) and ß-stiffness index] and platelet activation.

**Methods:**

In 43 PKU patients and in 58 healthy controls we prospectively examined the fatty acid profile, CIMT, ß-stiffness index and platelet activation (flow cytometric determination of markers of platelet activation). CIMT was measured bilaterally by ultrasound. CIMT _mean_ was defined as the mean value of the sum of CIMT _left_ and CIMT _right_.

**Results:**

Despite of lower HDL-cholesterol and higher triglyceride concentrations in the PKU group, there was no significant difference in the omega-6 or omega-3 fatty acid profile, CIMT, ß-stiffness index between both groups. Platelet activation was not enhanced in the PKU group.

**Conclusions:**

Fish-free diet does not induce early atherosclerotic changes or enhanced platelet activation in PKU patients.

## Introduction

Classical phenylketonuria is an inherited disease characterised by the absence or deficiency of the enzyme phenylalanine hydroxylase, resulting in an increase in blood phenylalanine concentration [1]. The aim of treatment is to lower blood phenylalanine concentration to prevent mental retardation [1]. The controlled low-phenylalanine diet is started in the first weeks of life and should be adhered to lifelong [2,3]. The low-phenylalanine diet consists of vegan-vegetarian fare (no meat, fish, milk, cheese, eggs, nuts, bread, or soya products) plus a synthetic, phenylalanine-free “medical food-formula” containing essential and nonessential amino acids, vitamins, trace elements, and minerals but without fat supplement [1,2]). As these patients never consume fish, this may result in a chronically omega-3 and omega-6 fatty acids deficiency with the risk of early atherosclerotic changes. Several epidemiological studies [4–10] show an association between cardiovascular disease and the consumed amount of omega-3 or omega-6 fatty acids. The atherothrombotic risk of adult PKU patients has not been investigated so far. Hence we analysed early atherosclerotic changes (surrogate markers as carotid media thickness and ß-stiffness index) in PKU patients in comparison to a healthy control group. Since platelets play a critical role in atherogenesis and its complications, we also investigated platelet activation in these patients, especially in the light of reports that platelets of vegetarians and vegans show hyperaggregability to agonists [11, 12, 13].

## Methods

### Patients

The study was conducted according to the principles of the Declaration of Helsinki and was approved by the local ethics committee Charite-Universitaetsmedizin Berlin. All participants provided written informed consent. Written informed consent was obtained and stored. The ethics approval number was EA2/089/05.

Forty-three PKU patients and 58 healthy controls were prospectively enrolled in the study. The inclusion of PKU patients was restricted to those with a lifelong phenylalanine-reduced diet i.e. patients diagnosed by newborn screening. In Germany screening for PKU has been initiated in 1970, therefore the maximum age of the patients investigated was 38 years. Compliance to the diet was controlled by measuring plasma phenylalanine and tyrosine levels of the PKU patients. The PKU patients were seen in regular intervals at our outpatient clinic.

### Ultrasound evaluation

#### Intima-Media thickness (IMT)

Ultrasonographic scanning was performed using an ultrasonic phase-locked echotracking system, which was equipped with a high-resolution real-time 8-MHz linear 2D scanner (Sytem Five, GE-Vingmed, Solingen Germany). The intima-media thickness (CIMT) was measured bilaterally at a defined location, 30 mm proximal to the bifurcation at the far wall of the common carotid artery (ACC) provided that this point was free of calcification. If there was an atherosclerotic calcified plaque the measurement was done 25 mm proximal to the bifurcation. CIMT_right_ and CIMT_left_ were determined and the average CIMT_mean_ was calculated. The investigator who performed the CIMT analysis was blinded to the results of the platelet measurements and the diet protocol of the patients.

#### β-Stiffness-Index

Several parameters have been proposed as indexes of the elastic properties of the arteries. In the present study we determined the arterial stiffness of both carotid arteries and calculated the average of the right and left β-stiffness index. The stiffness-index is based on the assumption that an exponential relation exists between relative pressure and strain [14]. Carotid stiffness index was calculated using the following formula: β = (natural logarithmic systolic blood pressure–natural logarithmic diastolic blood pressure)/ (systolic diameter–diastolic diameter)/diastolic diameter [15,16].

### Platelet analysis

For flow cytometric analysis 0.5 ml of blood was collected into a polypropylene syringe with 1.0 ml of a fixative containing methacrolein (CyfixII) [17]. Evaluation of surface expression of platelet membrane glycoproteins (CD62P, CD63, CD40L, PAC-1) was performed with specific monoclonal antibodies and two-color whole blood flow cytometry, as described in detail elsewhere [18]. Specific monoclonal antibody binding was expressed as mean immunofluorescence intensity (CD63, CD40L) or percentage of positive platelets of total platelet population (CD62P, PAC-1) and was used as a quantitative measurement of surface glycoprotein expression. The platelet assay used in the present study obtains reproducible results without significant artifactual platelet activation and has proved suitable for platelet analysis in a variety of clinical settings [19–21]. To establish reference values, control samples obtained from healthy individuals were always processed simultaneously with patient samples.

### Determination of fatty acid profile

Fatty acid and lipid profiles were measured in red cell membranes according to a modified procedure of Morrison et al [22] and Eder et al [23]. Lipid extraction was performed by the hexan-isopropanol extraction technique [24].

### Statistical analyses

Results of flow cytometric parameters are reported as median (interquartile range) unless otherwise indicated. Differences between groups were evaluated either by using the t-test for parameters showing normal distribution or by appropriate unpaired nonparametric tests (Mann Whitney U test) for parameters not normally distributed. A value of p<0.05 was regarded as significant (SPSS, Windows, version 10.0).

## Results

### Demographic data of the patients

Between April 2005 and November 2005 we consecutively enrolled 43 PKU patients and 58 healthy controls. The basic demographic and clinical characteristics are given in **Table 1**. The PKU group comprised less women (48.8%) than the control group (65.5%) and the body mass index of the PKU patients was significantly greater (24.30 ± 0.69 kg/m² versus 21.96 ± 0.31 kg/m²; p = 0.009). HDL-cholesterol was significantly lower in the PKU group compared to the controls (51.8 ± 2.43 mg/dl versus 63.11 ± 2.23 mg/dl; p = 0.003), but still in a normal range, whereas there was no difference in total cholesterol and LDL-cholesterol. The remaining routine clinical and laboratory parameters were not statistically different between both groups. Compliance to the diet was controlled by measuring plasma phenylalanine and tyrosine levels of the PKU patients. In our patient group plasma phenylalanine was 15.5 ± 0.6 mg/dl and tyrosine 0.9 ± 0.08 mg/dl respectively.

**Table 1:** Baseline characteristics of the patients and controls.

Patients	PKU n = 43	Controls n = 58	p-value
Age, years	28.1 ± 0.96	26.2 ± 0.5	0.434
Female gender, n (%)	21 (48.8)	39 (67.2)	0.064
Smokers, n (%)	8 (18.6)	14 (24.2)	0.546
Body mass index, kg/m2	24.3 ± 0.7	21.9 ± 0.3	0.009
Platelets, nl^-1^	255 ± 10.2	239 ± 6.7	0.167
Leukocytes, nl^-1^	6.8 ± 0.3	5.9 ± 0.2	0.031
Cholesterol, mg/dl	160 ± 4.8	168 ± 4.3	0.167
LDL-cholesterol, mg/dl	84.3 ± 4.2	89.9 ± 3.8	0.374
HDL-cholesterol, mg/dl	51.8 ± 2.4	63.1 ± 2.2	0.003
Triglycerides, mg/dl	108.7± 8.9	88.2± 5.3	0.017

Results are shown as mean ± SEM or n (%)

### Intima media thickness and β-stiffness index

**Fig 1:**
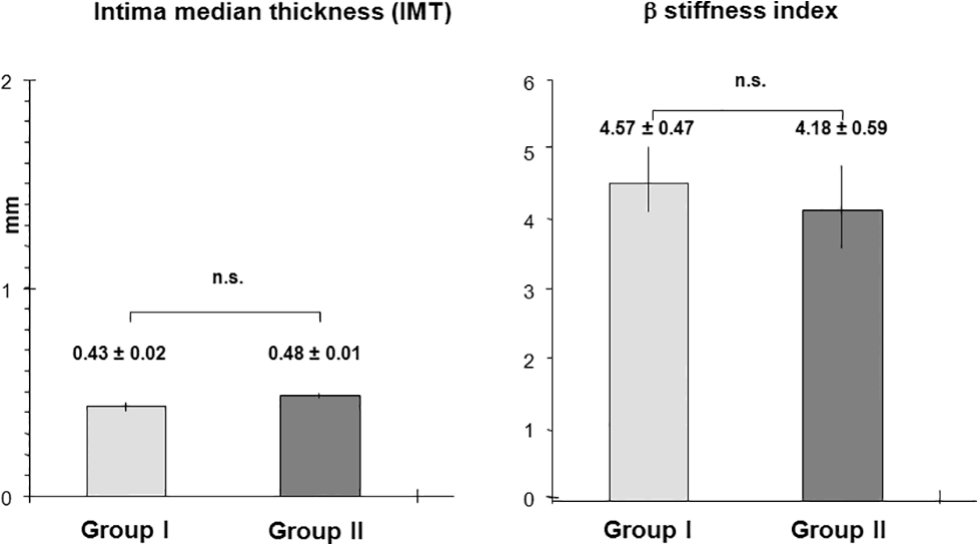
Schematic representation of carotid intima media thickness (CIMT) and β-stiffness index in PKU patients (group I) and controls (group II). Bar-graphs showing the mean value and SEM. There was no significant difference in CIMT [(0.43 ± 0.02 mm) versus (0.48 ± 0.01mm), p_CIMT_ = 0.88] and β-stiffness index [(4.57 ± 0.47) versus 4.18 ± 0.59) p_ß_ = 0.7].

The surrogate markers CIMT and β-stiffness index were not significantly different between the PKU patients [(CIMT: 0.43 ± 0.02 mm; β = 4.57 ± 0.47) and the healthy controls (CIMT: 0.48 ± 0.01 mm; β = 4.18 ± 0.59) p_CIMT_ = 0.88 and p_β_ = 0.70] **(Fig 1).**

### Platelet markers

**Fig 2:**
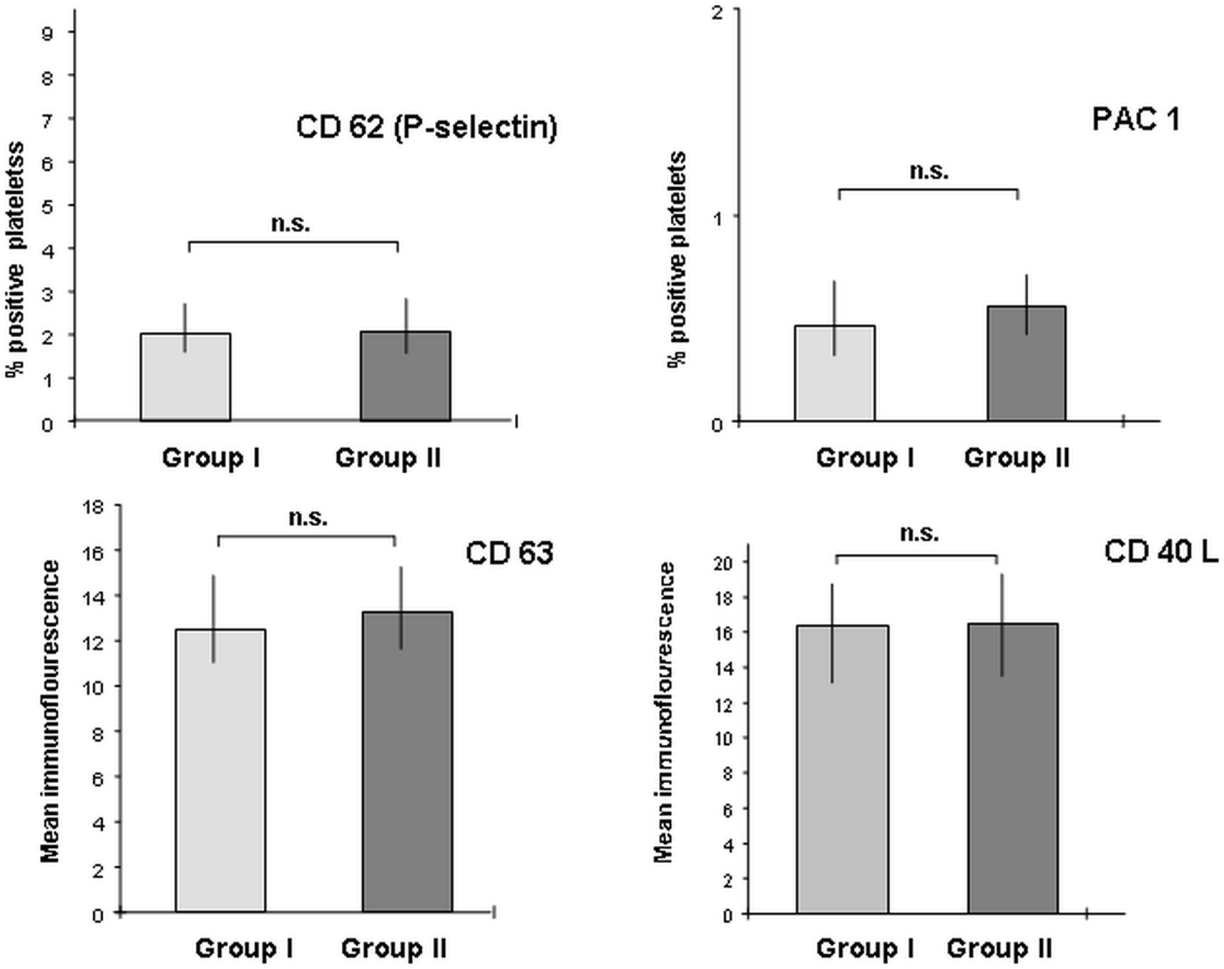
Schematic expression of platelet activation markers CD62P, PAC-1, CD63 and CD40 L in PKU patients (group I) versus controls (group II). Histobars representing a) percentage of positive platelets as [median; interquartile range]: CD62P: [2.0; 1.6–2.7] versus [2.1; 1.57–2.8], p = 0.86 and PAC-1: [0.46; 0.32–0.68] versus [0.55; 0.42–0.71], p = 0.29; b) Mean immunofluorescence intensity (MFI); [median; interquartile range]: CD63 [12.5; 11.0–14.9] versus [13.23; 11.61–15.25], p = 0.3; and CD40L [16.31; 13.10–18.7] versus [16.5; 13.5–19.3], p = 0.62.

Surface expression of activation dependent platelet membrane glycoproteins (CD62P, PAC-1, CD63 and CD40L) was not enhanced in the PKU group compared to the control group: CD62 P (percentage of positive platelets) as [median; interquartile range] in the PKU group versus control group: [(2.0; 1.6–2.7) versus (2.1; 1.57–2.8), p = 0.86] and PAC-1 (percentage of positive platelets) as [median; interquartile range]: [(0.46; 0.32–0.68) versus (0.55; 0.42–0.71), p = 0.29] (Fig 2A).

CD63 (mean immunofluorescence intensity) as [median; interquartile range]: [12.5; 11.0–14.9] versus [13.23; 11.61–15.25], p = 0.3 and CD40L (mean immunofluorescence intensity) as [median; interquartile range]: [16.31; 13.1–18.7] versus [16.5; 13.5–19.3], p = 0.62 (Fig 2B).

### Fatty acid profile

The level of omega-6 fatty acids and surprisingly also of the omega-3 fatty acids in red cell membranes did not differ significantly between the PKU and the control group **(Table 2).** The content of omega -3 fatty acids was 8.22 ± 24 amount % in PKU patients and 8.41 ± 24 amount % in the control group. We also found no significant difference when we further discriminated the omega-3 fatty acids in the following chains 18:3, 20:5, 22:5 and 22:6. (**Table 2**). The concentration of omega-6 fatty acids was 26.39 ± 1.29 amount % in the PKU group and 26.61 ± 1.08 amount % in the control group. Concerning the content of the omega-6 fatty acid chains 20:3 and 20:4 there was also no significant difference between both groups. The omega-3/omega-6 ratio was also not significant different between both groups (1:3.3 versus controls: 1:3.26; p = 0.54).

**Table 2:** Fatty acid profile.

Patients	PKU n = 43	Controls n = 58	p-value
**Omega-3 fatty acids**, amount %	8.22 ± 0.24	8.41 ± 0.22	0.52
α-Linolenic acid (ALA), 18:3	0.20 ± 0.03	0.19 ± 0.02	0.995
Eicosapentaenoic acid (EPA), 20:5	0.59 ± 0.04	0.62 ± 0.04	0.686
Docosapentapentaenoic acid (DPA), 22:5	1.98 ± 0.14	1.95 ± 0.12	0.816
Dococohexaenoic acid (DHA), 22:6	4.20 ± 0.15	4.50 ± 0.17	0.167
**Omega-6 fatty acids, amount %**	26.39 ± 1.29	26.61 ± 1.08	0.94
Dihomo-gamma-linoleic acid, 20:3	1.42 ± 0.08	1.34 ± 0.07	0.331
Arachidonic acid, 20:4	11.43 ± 0.64	11.38 ± 0.07	0.959
Omega-3/omega-6 fatty acid ratio	1:3.3 ± 0.19	1:3.26 ± 0.17	0.54

Results are shown as mean ± SEM; p value of < 0.05 was regarded as significant *.

Fatty acid profile were measured in red cell membranes.

## Discussion

This is the first study to analyse the risk for early atherosclerotic changes (surrogate marker CIMT and β-stiffness index) and platelet activation in adult PKU patients. Although PKU patients keeping to a life-long phenylalanine-restricted diet, never eat fish, we did not find early atherosclerotic changes or enhanced platelet activation. The total amount of omega-3 and omega-6 fatty acids and likewise also the ratio of omega-3 /omega-6 fatty acids did not differ significantly between the PKU group and the controls. These results were not due to lack of compliance of diet since phenylalanine blood levels were always within recommended limits, respectively 0.7–20 mg/dl. Surprisingly the PKU patients showed even no deficiency of the omega-3 fatty acid chains as 18:3, 20:5 and 22:5 although the intake of these fatty acids is mostly by fatty fish. It seems that PKU patients compensate successfully the lack of omega-3 fatty acid intake caused by fish-free diet by the intake of special vegetable oils as for instance canola oil or flax seed oil. On the other hand one might argue that there was no difference because the intake of fatty fish is also low in the healthy control group.

Our findings are in contrast to Mosely [25] who in 2002 found significant lower levels of omega-3 fatty acids in adult PKU patients. In contrast to earlier (before 2002) nutritional recommendations, PKU patients today are encouraged to balance their diet by vegetable fats rich in omega-3 fatty acids. As a result of the predominantly vegetable fat intake by PKU patients, we observed significantly lower levels of saturated fatty acids compared to the control group.

Pioneering studies in Greenland Eskimos almost 30 years ago suggested that ingestion of omega-3 fatty acids (mostly present in fatty fish) conveys protection from cardiovascular disease [4]. This initial observation has set off of a number of epidemiological studies to examine this hypothesis, but data from randomized trials are limited [26, 27] and case-control studies on fish intake and fatal CAD are sparse [5, 26, 27, 28]. Results from prospective cohort studies have been inconsistent. Some studies showed an inverse association between fish intake [8, 9, 10] and CAD mortality whereas others did not [29–30].

Possible mechanisms underlying the cardioprotective effects of the consumption of fish include the reduction in serum triglycerides, a decrease in platelet aggregability and the presence of antiarrhythmic effects [31]. But it is still not clear whether omega-3 fatty acids have a direct effect on the pathogenesis of atherosclerosis. The addition of omega-3 fatty acids to standard diets demonstrated no protective effect on the progression of atherosclerosis in carotid arteries [31, 32]. A large study including 551 patients, failed to show that supplementation of 8 g/d of omega-3 fatty acids prevent restenosis following angioplasty [33]. Consequently, at present, we have no evidence that the intake of omega-3 fatty acids induce regression of atherosclerosis although the beneficial effect in reducing mortality after a first myocardial infarction has been demonstrated in many studies [34]. One might speculate that although omega-3 fatty acids have been demonstrated to prevent cardiac death in patients after myocardial infarction, they exhibit no direct effect on the early stages of atherosclerosis. The latter could explain why there was no difference in carotid intima-media thickness and β-stiffness-index between both groups.

Data concerning the relation between platelet function and omega-3 and omega-6 fatty acid levels are controversial. Dyerberg and Bang [4] first reported on the decreased platelet function in Greenlanders. Since that seminal observation, data have been aquired to provide support for several mechanisms whereby omega-3 fatty acids help balance cellular-signalling networks to more antithrombotic, anti-inflammatory set points. Omega-3 fatty acids competewith and antagonize the metabolism of omega-6 fatty acids and eicosanoids [35]. Eicosanoids derived from omega-3 fatty acids are less efficient amplifiers of the cell-signalling process in contrast to those derived from the omega-6 fatty acid, arachidonic acid. Despite this reasonable mechanism of omega-3 fatty acids, intervention studies with omega-3 polyunsaturated fatty acids gave conflicting results. Mori [36] showed significant reduction of platelet aggregation to agonists as collagen and platelet activating factor (PAF) after supplementation with fish and /or fish oil. Prisco [37] demonstrated also decreased collagen-induced platelet aggregation in patients receiving omega-3 fatty acid supplementation. Braden [38] reported a modest platelet inhibitory effect of fish oil in vivo in a canine model of coronary thrombosis. However there exist also other studies [39, 40, 41] showing no effect of omega-3 supplementation on platelet aggregation and thromboxan production.

Platelets of vegetarians or vegans are said to be more sensitive to proaggregatory agonists than those of omnivores [42]. Suboptimal taurine status [42] besides deficiency of omega-3 fatty acids in vegans is reported to be responsible for the increased aggregability of platelets. Thus platelet activation in PKU patients could occur independently from the lipid status. Yet, the expression of platelet activation markers (CD62P, PAC-1, CD40L and CD63) in our PKU patients was not enhanced in comparison to the control group. But PKU patients on phenylalanine-restricted diet are strictly speaking no real vegans, because their diet include the intake of synthetic, phenylalanine-free “medical food-formula” containing essential and non-essential amino acids, including taurine, vitamins, trace elements, and minerals.

Our data can be seen in the light of the Origin-trial [43] investigating the addition of n-3 fatty acids vs placebo on the overall cardiovascular outcome in a high-risk patient group. This trial demonstrated no positive effect of the addition of n-3 fatty acids on the overall cardiovascular outcome. Our results may help to further clarify the role of fish oil and its effect on cardiovascular outcome. By means of this specific group of patients who besides the nutritional difference of never eating fish are well comparable to the normal population, we could demonstrate that the lack of fish oil has no negative effect on early atherosclerotic changes and platelet activation, despite the higher BMI, triglyceride and the lower HDL cholesterol concentrations. Hence these data challenge some traditional beliefs that i.e the lack of unsaturated fatty acids as they are contained in fish meals may have a negative effect on early atherosclerotic changes. The PKU group is the only group of patients who never eats fish and has the great advantage that we can monitor compliance of diet by measuring the phenylalanine blood concentrations. In all other studies one has to rely on the uncertainty of anamnestic statements to eating habits.

## Conclusion

According to our results, fish free diet does not induce early atherosclerotic changes and platelet activation in PKU patients. Surprisingly omega-3 fatty acid level were not different in both groups implicating that PKU patients compensate their lack of fish intake by vegetable fats rich in omega-3 fatty acids. Further interventional studies with omega-3 supplementation have to evaluate the long-term effect on atherosclerotic surrogate markers and platelet function in these patients.
